# Supplementary material for: Machine learning predicts upper secondary education dropout as early as the end of primary school
Source: Sci Rep. 2024 Jun 5;14:12956. doi: 10.1038/s41598-024-63629-0 (PMC11153526; doi:10.1038/s41598-024-63629-0)
Supplement: Supplementary file 1 — Supplementary Information. [file 41598_2024_63629_MOESM1_ESM.docx]

**Supplementary Information**

**Table S1**

Feature scores for the B-RandomForest models across the six-fold cross-validation with data up to Grade 9.

| Feature Code^a^ | Score |
| --- | --- |
| READ2 | 0.016734 |
| READ4 | 0.016591632803953937 |
| READ3 | 0.015962487876521644 |
| READ1 | 0.015671323157721368 |
| RAN | 0.015174431545370501 |
| multSC7 | 0.014838193357266202 |
| ariSC4 | 0.014376882154063741 |
| ly1C5c | 0.012205 |
| ariSC6 | 0.012174298632531026 |
| ly4C7c | 0.011741475047299164 |
| ly1C4c | 0.011671491394929236 |
| ariSC7 | 0.011593380287125908 |
| ariSC5 | 0.011526 |
| ppvSC2 | 0.010803 |
| pisaC10total_sum | 0.010392 |
| ariSC3 | 0.010059813904038421 |
| multSC9 | 0.009841 |
| READ9 | 0.00982 |
| READ7 | 0.009386 |
| ly1C6c | 0.009132 |
| READ6 | 0.009026 |
| multSC11 | 0.008987 |
| ravSC6 | 0.008883 |
| voedo | 0.008646 |
| ly7C9c | 0.008602 |
| msqSC2 | 0.008602 |
| multSC8 | 0.008583 |
| tavma_G9 | 0.007201 |
| behen_G9 | 0.007091 |
| tavli_G6 | 0.006906 |
| ly7C11c | 0.006763 |
| tavma_G6 | 0.006528 |
| tavli_G7 | 0.00616 |
| buexh_G9 | 0.006151 |
| ariSC9 | 0.00615 |
| selfma_G9 | 0.006134 |
| ariSC11 | 0.005943 |
| tavma_G7 | 0.005909 |
| schwo_G9 | 0.005839 |
| ariSC8 | 0.005804 |
| ly6C8c | 0.005705 |
| tavli_G9 | 0.005644 |
| behen_G7 | 0.005456 |
| buexh_G7 | 0.005297 |
| rses_G6 | 0.005219 |
| emot_G9 | 0.0052 |
| bucyn_G7 | 0.005121 |
| buexh_G6 | 0.005115 |
| bucyn_G9 | 0.005106 |
| behen_G6 | 0.005092 |
| schwo_G7 | 0.005037 |
| hesmoC11 | 0.004993 |
| selfma_G6 | 0.004958 |
| peersup_G9 | 0.004947 |
| emot_G7 | 0.004938 |
| hyper_G9 | 0.004932 |
| rses_G9 | 0.00487 |
| prosoc_G6 | 0.004802 |
| peerp_G9 | 0.004798 |
| bucyn_G6 | 0.004732 |
| hyper_G6 | 0.004718 |
| rses_G7 | 0.004579 |
| selfli_G6 | 0.004454 |
| emot_G6 | 0.004424 |
| testre_G9 | 0.00436 |
| prosoc_G9 | 0.004324 |
| hyper_G7 | 0.004291 |
| selfma_G7 | 0.004289 |
| schbe_G9 | 0.004158 |
| prosoc_G7 | 0.004148 |
| inter3C9 | 0.004074 |
| inter2C8 | 0.004066 |
| abs2C11 | 0.004056 |
| claeng_G9 | 0.004039 |
| schbe_G7 | 0.004031 |
| testre_G7 | 0.00401 |
| selfli_G7 | 0.003904 |
| selfli_G9 | 0.003806 |
| peerp_G7 | 0.003801 |
| inter12C9 | 0.0038 |
| inter1C11 | 0.003582 |
| peersup_G7 | 0.003581 |
| peerp_G6 | 0.003573 |
| inter23C11 | 0.003472 |
| future_G9 | 0.003427 |
| inter3C11 | 0.003401 |
| inter3C8 | 0.003388 |
| difmatC11 | 0.003363 |
| inter20C9 | 0.003357 |
| difmataC9 | 0.003356 |
| future_G7 | 0.003316 |
| claeng_G7 | 0.003306 |
| cprob_G9 | 0.003305 |
| med2C9 | 0.003295 |
| healcC11 | 0.00326 |
| inter12C11 | 0.003252 |
| mob1C8 | 0.003226 |
| inter4C8 | 0.003203 |
| cprob_G7 | 0.003177 |
| med3C9 | 0.003168 |
| inter1C8 | 0.003156 |
| inter11C8 | 0.003141 |
| inter15C9 | 0.003133 |
| inter20C8 | 0.003111 |
| mob1C9 | 0.003106 |
| inter2C11 | 0.003102 |
| cprob_G6 | 0.003092 |
| inter8C8 | 0.00308 |
| med4C8 | 0.00306 |
| difmatC9 | 0.002987 |
| chgenderC1C13 | 0.002971 |
| inter9C8 | 0.002953 |
| mob1priC11 | 0.002917 |
| familysup_G9 | 0.002915 |
| inter1C9 | 0.002906 |
| inter17C11 | 0.00289 |
| hesmoC9 | 0.002861 |
| inter9C9 | 0.002854 |
| inter10C8 | 0.002853 |
| difmataC11 | 0.002847 |
| inter23C8 | 0.002808 |
| familysup_G7 | 0.002772 |
| inter8C9 | 0.002765 |
| diflitaC9 | 0.002751 |
| inter13C9 | 0.002734 |
| diflitaC11 | 0.002706 |
| inter5C8 | 0.002694 |
| med2C11 | 0.002651 |
| difmataC8 | 0.00262 |
| inter24C11 | 0.00262 |
| inter2C9 | 0.002606 |
| med2C8 | 0.002602 |
| diflitaC8 | 0.00259 |
| med4C9 | 0.002574 |
| med3C11 | 0.002572 |
| med4C11 | 0.002555 |
| inter12C8 | 0.002546 |
| inter22C8 | 0.002537 |
| inter13C8 | 0.00253 |
| inter15C8 | 0.002505 |
| inter4C9 | 0.0025 |
| inter13C11 | 0.002475 |
| inter8C11 | 0.002473 |
| ses_ses4.0 | 0.002468 |
| inter5C9 | 0.002385 |
| inter22C9 | 0.002372 |
| mob2C8 | 0.002365 |
| med5C8 | 0.002328 |
| pubonset | 0.002323 |
| mob1pubC11 | 0.002311 |
| med1C8 | 0.002299 |
| inter18C8 | 0.00229 |
| med1C9 | 0.002279 |
| ses_ses3.0 | 0.00227 |
| inter9C11 | 0.002263 |
| med1C11 | 0.002246 |
| med3C8 | 0.00221 |
| inter18C9 | 0.002206 |
| inter4C11 | 0.002168 |
| famstruct_fs1.0 | 0.002159 |
| inter21C11 | 0.00212 |
| famstruct_fs3.0 | 0.002104 |
| inter14C9 | 0.002081 |
| bullfrC8 | 0.002074 |
| inter25C11 | 0.002058 |
| inter14C8 | 0.002052 |
| inter11C9 | 0.002047 |
| inter16C11 | 0.002035 |
| famstruct_fs2.0 | 0.002028 |
| inter26C8 | 0.002028 |
| inter23C9 | 0.001986 |
| inter26C9 | 0.001958 |
| inter21C8 | 0.001957 |
| inter14C11 | 0.001955 |
| abs2C9 | 0.00193 |
| geatmaF2dC9_gma2d91.0 | 0.00192 |
| inter20C11 | 0.001895 |
| inter5C11 | 0.001886 |
| inter21C9 | 0.001885 |
| inter19C11 | 0.001881 |
| inter30C11 | 0.001841 |
| inter19C8 | 0.001782 |
| plan_aC9rec | 0.001745 |
| geatmaF2dC9_gma2d90.0 | 0.001697 |
| ses_ses1.0 | 0.001628 |
| famstruct_fs4.0 | 0.00162 |
| acctypeC9_acc91.0 | 0.001541 |
| inter18C11 | 0.001539 |
| hesmoC8 | 0.001521 |
| acctypeC8_acc81.0 | 0.001521 |
| geatmaF1bC8_gma1b80.0 | 0.001493 |
| geatmaF1bC8_gma1b81.0 | 0.001484 |
| inter26C11 | 0.00148 |
| inter6C8 | 0.001467 |
| geatliF1aC11_g1a111.0 | 0.001447 |
| inter15C11 | 0.001443 |
| bullfrC9 | 0.001413 |
| difreaC11 | 0.001409 |
| acctypeC11_acc111.0 | 0.001399 |
| healcC9 | 0.00139 |
| inter17C8 | 0.001361 |
| inter10C9 | 0.001353 |
| geatmaF2dC11_gma2d111.0 | 0.001347 |
| inter17C9 | 0.001322 |
| diflitC9 | 0.001297 |
| inter24C8 | 0.001282 |
| plan_aC11rec | 0.001256 |
| geatliF2dC9_g2d90.0 | 0.00124 |
| geatmaF2bC11_gma2b110.0 | 0.00124 |
| inter19C9 | 0.001222 |
| inter11C11 | 0.001213 |
| geatmaF2dC11_gma2d110.0 | 0.001211 |
| geatliF1aC11_g1a110.0 | 0.001207 |
| geatliF2bC9_g2b91.0 | 0.00118 |
| geatmaF2dC8_gma2d81.0 | 0.001156 |
| healcC8 | 0.001142 |
| inter16C8 | 0.001129 |
| geatliF2cC9_g2c91.0 | 0.001114 |
| geatmaF2bC9_gma2b91.0 | 0.00111 |
| bullfrC11 | 0.001106 |
| geatmaF2cC9_gma2c91.0 | 0.001098 |
| geatliF1bC8_g1b80.0 | 0.001083 |
| geatmaF1dC8_gma1d80.0 | 0.001082 |
| geatmaF2bC9_gma2b90.0 | 0.001074 |
| acctypeC8_acc82.0 | 0.001064 |
| bullseC9 | 0.001061 |
| geatliF1bC8_g1b81.0 | 0.001057 |
| ses_ses5.0 | 0.001054 |
| bullseC8 | 0.001052 |
| geatliF2bC8_g2b80.0 | 0.001051 |
| geatliF2dC8_g2d81.0 | 0.001048 |
| geatliF2bC8_g2b81.0 | 0.001045 |
| buintC9 | 0.001045 |
| geatliF2dC9_g2d91.0 | 0.001035 |
| geatmaF1bC9_gma1b91.0 | 0.001035 |
| geatliF1bC11_g1b110.0 | 0.001027 |
| geatmaF2cC9_gma2c90.0 | 0.001025 |
| geatmaF2bC8_gma2b80.0 | 0.00102 |
| geatmaF2bC11_gma2b111.0 | 0.001019 |
| inter28C11 | 0.001014 |
| geatmaF1aC11_gma1a110.0 | 0.001014 |
| bullseC11 | 0.001013 |
| geatliF1dC8_g1d80.0 | 0.001012 |
| geatliF1dC8_g1d81.0 | 0.001007 |
| geatliF2bC11_g2b110.0 | 0.001004 |
| geatmaF1bC11_gma1b111.0 | 0.000998 |
| geatliF2dC11_g2d110.0 | 0.000982 |
| geatmaF1dC9_gma1d90.0 | 0.000982 |
| geatliF1bC9_g1b91.0 | 0.000973 |
| geatliF1bC11_g1b111.0 | 0.000972 |
| inter22C11 | 0.000967 |
| inter29C11 | 0.000966 |
| geatliF2dC11_g2d111.0 | 0.00096 |
| geatliF1dC11_g1d111.0 | 0.000949 |
| geatmaF1dC8_gma1d81.0 | 0.000944 |
| geatliF2cC9_g2c90.0 | 0.000941 |
| geatmaF1dC11_gma1d110.0 | 0.000941 |
| geatmaF2cC8_gma2c80.0 | 0.000932 |
| geatliF1cC8_g1c81.0 | 0.000926 |
| geatmaF1bC11_gma1b110.0 | 0.00092 |
| geatmaF2cC8_gma2c81.0 | 0.000918 |
| geatmaF1cC9_gma1c91.0 | 0.000907 |
| geatmaF2bC8_gma2b81.0 | 0.000904 |
| geatliF2bC11_g2b111.0 | 0.0009 |
| geatliF2aC11_g2a111.0 | 0.000895 |
| geatmaF2aC11_gma2a111.0 | 0.000893 |
| inter25C8 | 0.000893 |
| geatmaF1cC8_gma1c80.0 | 0.00089 |
| inter7C8 | 0.000887 |
| geatmaF2dC8_gma2d80.0 | 0.000882 |
| geatliF2aC11_g2a110.0 | 0.000871 |
| buintC11 | 0.000869 |
| geatmaF1cC9_gma1c90.0 | 0.000869 |
| bumobC8 | 0.000869 |
| geatliF2cC11_g2c110.0 | 0.000869 |
| acctypeC11_acc112.0 | 0.000866 |
| geatliF1dC11_g1d110.0 | 0.000853 |
| buintC8 | 0.000853 |
| inter6C9 | 0.000846 |
| geatliF2dC8_g2d80.0 | 0.000846 |
| geatliF2bC9_g2b90.0 | 0.000841 |
| geatmaF1cC8_gma1c81.0 | 0.000838 |
| inter25C9 | 0.000832 |
| geatliF1cC8_g1c80.0 | 0.000821 |
| bumobC11 | 0.000818 |
| bumobC9 | 0.000809 |
| geatmaF1dC11_gma1d111.0 | 0.000793 |
| geatliF2cC11_g2c111.0 | 0.000788 |
| inter10C11 | 0.000787 |
| geatmaF2cC11_gma2c110.0 | 0.000787 |
| geatmaF2aC11_gma2a110.0 | 0.000781 |
| geatmaF1dC9_gma1d91.0 | 0.000773 |
| geatliF1bC9_g1b90.0 | 0.00077 |
| geatmaF1cC11_gma1c110.0 | 0.000767 |
| geatmaF1bC9_gma1b90.0 | 0.00076 |
| acctypeC8_acc84.0 | 0.000756 |
| acctypeC9_acc94.0 | 0.000753 |
| geatliF1dC9_g1d90.0 | 0.00073 |
| geatliF1cC9_g1c90.0 | 0.000729 |
| geatliF1aC9_g1a91.0 | 0.000728 |
| abs2C8 | 0.00072 |
| geatliF2cC8_g2c81.0 | 0.000713 |
| geatliF1cC11_g1c110.0 | 0.000695 |
| geatliF2cC8_g2c80.0 | 0.000688 |
| geatmaF2cC11_gma2c111.0 | 0.000681 |
| geatliF1aC8_g1a80.0 | 0.000679 |
| geatliF1cC11_g1c111.0 | 0.000676 |
| geatliF2aC8_g2a81.0 | 0.000676 |
| geatliF2aC9_g2a90.0 | 0.000673 |
| inter24C9 | 0.000666 |
| geatmaF1aC11_gma1a111.0 | 0.000651 |
| geatmaF2aC9_gma2a91.0 | 0.000646 |
| geatliF1dC9_g1d91.0 | 0.000642 |
| geatliF2aC8_g2a80.0 | 0.000626 |
| buintsC9 | 0.000623 |
| geatmaF2aC8_gma2a80.0 | 0.000617 |
| buintsC8 | 0.000602 |
| geatliF1aC8_g1a81.0 | 0.000598 |
| geatmaF1aC9_gma1a90.0 | 0.000592 |
| acctypeC9_acc92.0 | 0.00059 |
| acctypeC11_acc114.0 | 0.000582 |
| inter16C9 | 0.00058 |
| geatmaF1aC9_gma1a91.0 | 0.000567 |
| bumobsC8 | 0.000566 |
| geatliF1aC9_g1a90.0 | 0.000559 |
| inter7C9 | 0.000559 |
| geatmaF2aC8_gma2a81.0 | 0.000554 |
| acctypeC8_acc85.0 | 0.000551 |
| acctypeC9_acc95.0 | 0.000549 |
| bumobsC9 | 0.000547 |
| geatmaF1aC8_gma1a80.0 | 0.000547 |
| geatliF2aC9_g2a91.0 | 0.000546 |
| geatmaF2aC9_gma2a90.0 | 0.000539 |
| geatliF1cC9_g1c91.0 | 0.000532 |
| geatmaF1aC8_gma1a81.0 | 0.000527 |
| geatmaF1cC11_gma1c111.0 | 0.000526 |
| acctypeC11_acc115.0 | 0.000509 |
| inter6C11 | 0.00045 |
| inter7C11 | 0.00043 |
| inter27C11 | 0.000397 |
| bumobsC11 | 0.000347 |
| buintsC11 | 0.000341 |
| famstruct_fs5.0 | 0.000286 |
| acctypeC11_acc113.0 | 0.000241 |
| ses_ses7.0 | 0.000219 |
| famstruct_fs6.0 | 0.000217 |
| acctypeC9_acc93.0 | 0.000184 |
| acctypeC9_acc96.0 | 0.000117 |
| acctypeC8_acc83.0 | 0.000115 |
| acctypeC8_acc86.0 | 8.887077927021146e-05 |
| acctypeC11_acc116.0 | 6.264400399910738e-05 |
| acctypeC9_acc97.0 | 5.742113750216562e-05 |
| acctypeC9_acc98.0 | 4.9312457890394395e-05 |
| acctypeC8_acc87.0 | 4.6708425441638035e-05 |
| ses_ses8.0 | 3.646770546930522e-05 |
| acctypeC8_acc88.0 | 3.425778336748851e-05 |
| acctypeC11_acc117.0 | 2.439164494365861e-05 |
| ses_ses6.0 | 1.9773085109194213e-05 |
| acctypeC11_acc118.0 | 1.8591258543921192e-05 |

^a^ For detailed explanations of each feature code, refer to Table S3. Codes for features can be found in the 'Modalities' and 'Assessment Grade' columns.

**Table S2**

Feature scores for the B-RandomForest models across the six-fold cross-validation with data up to Grade 9.

| Feature Code^a^ | Scores |
| --- | --- |
| READ1 | 0.02784 |
| READ2 | 0.027707187734627808 |
| READ4 | 0.027643 |
| multSC7 | 0.02581 |
| READ3 | 0.025342960578520018 |
| RAN | 0.024715886705463843 |
| ariSC4 | 0.022619764239472328 |
| multSC8 | 0.022024 |
| READ6 | 0.021135 |
| ariSC6 | 0.019258 |
| ly1C5c | 0.019215 |
| ariSC7 | 0.019185858148111218 |
| ariSC5 | 0.019007832822908115 |
| voedo | 0.017464573237062107 |
| ly4C7c | 0.017315266493928894 |
| ly1C4c | 0.016856841619531677 |
| ppvSC2 | 0.01682 |
| tavma_G6 | 0.016634782666643946 |
| ariSC3 | 0.016266806714576584 |
| ly6C8c | 0.015993 |
| ariSC8 | 0.015481 |
| tavli_G6 | 0.015365549591987543 |
| ly1C6c | 0.014702370667713757 |
| ravSC6 | 0.013678492250656387 |
| behen_G6 | 0.013545787190862676 |
| rses_G6 | 0.013311983706013344 |
| selfma_G6 | 0.012769434834571223 |
| buexh_G6 | 0.01276 |
| bucyn_G6 | 0.012668 |
| hyper_G6 | 0.01249 |
| msqSC2 | 0.012486254957709575 |
| prosoc_G6 | 0.011878164119779369 |
| selfli_G6 | 0.011825129383668717 |
| emot_G6 | 0.011713380934920517 |
| pubonset | 0.011211 |
| peerp_G6 | 0.009449 |
| cprob_G6 | 0.009117 |
| mob1C8 | 0.008956 |
| inter2C8 | 0.008923 |
| inter1C8 | 0.008908 |
| inter12C8 | 0.008513 |
| inter9C8 | 0.008463 |
| inter3C8 | 0.008391 |
| inter8C8 | 0.008215 |
| chgenderC1C13 | 0.008039 |
| inter20C8 | 0.007951 |
| med2C8 | 0.007864 |
| inter5C8 | 0.007762 |
| inter15C8 | 0.007525 |
| med4C8 | 0.007503 |
| inter4C8 | 0.007294 |
| difmataC8 | 0.007282 |
| med1C8 | 0.007281 |
| diflitaC8 | 0.00701 |
| inter11C8 | 0.00696 |
| med5C8 | 0.006616 |
| inter10C8 | 0.006432 |
| mob2C8 | 0.006386 |
| inter18C8 | 0.006376 |
| inter13C8 | 0.006336 |
| inter23C8 | 0.006213 |
| inter22C8 | 0.006196 |
| bullfrC8 | 0.005566 |
| hesmoC8 | 0.005414 |
| inter26C8 | 0.005265 |
| med3C8 | 0.004974 |
| inter14C8 | 0.004805 |
| inter21C8 | 0.004699 |
| inter19C8 | 0.004509 |
| acctypeC8_acc81.0 | 0.004246 |
| ses_ses3.0 | 0.004004 |
| famstruct_fs1.0 | 0.003924 |
| geatmaF1bC8_gma1b80.0 | 0.003815 |
| ses_ses4.0 | 0.003623 |
| bullseC8 | 0.003604 |
| inter24C8 | 0.003476 |
| famstruct_fs2.0 | 0.003441 |
| healcC8 | 0.003334 |
| geatmaF1bC8_gma1b81.0 | 0.003245 |
| inter17C8 | 0.003198 |
| geatmaF2bC8_gma2b81.0 | 0.003169 |
| inter6C8 | 0.003067 |
| famstruct_fs3.0 | 0.003054 |
| geatliF2bC8_g2b80.0 | 0.002938 |
| famstruct_fs4.0 | 0.002887 |
| geatliF2bC8_g2b81.0 | 0.002873 |
| geatmaF2bC8_gma2b80.0 | 0.002864 |
| inter7C8 | 0.002806 |
| geatliF1bC8_g1b81.0 | 0.002732 |
| acctypeC8_acc82.0 | 0.002682 |
| buintC8 | 0.002646 |
| ses_ses1.0 | 0.002574 |
| geatmaF1dC8_gma1d80.0 | 0.002567 |
| acctypeC8_acc84.0 | 0.002527 |
| geatliF1bC8_g1b80.0 | 0.002505 |
| bumobC8 | 0.002482 |
| geatliF1dC8_g1d80.0 | 0.00244 |
| geatmaF2dC8_gma2d81.0 | 0.002423 |
| geatliF2dC8_g2d80.0 | 0.00235 |
| inter25C8 | 0.00231 |
| geatmaF2dC8_gma2d80.0 | 0.002304 |
| geatliF2dC8_g2d81.0 | 0.002288 |
| geatliF2cC8_g2c81.0 | 0.002268 |
| inter16C8 | 0.002266 |
| geatmaF1cC8_gma1c81.0 | 0.002219 |
| geatmaF1dC8_gma1d81.0 | 0.002158 |
| geatmaF2cC8_gma2c80.0 | 0.002094 |
| geatliF1cC8_g1c81.0 | 0.002093 |
| geatliF1cC8_g1c80.0 | 0.002072 |
| geatliF1dC8_g1d81.0 | 0.002069 |
| geatliF2cC8_g2c80.0 | 0.002003 |
| abs2C8 | 0.001928 |
| geatmaF2cC8_gma2c81.0 | 0.001887 |
| geatmaF1cC8_gma1c80.0 | 0.001886 |
| geatliF2aC8_g2a81.0 | 0.001854 |
| geatliF1aC8_g1a81.0 | 0.001793 |
| ses_ses5.0 | 0.001695 |
| geatliF1aC8_g1a80.0 | 0.00165 |
| geatliF2aC8_g2a80.0 | 0.001602 |
| geatmaF2aC8_gma2a81.0 | 0.00156 |
| geatmaF2aC8_gma2a80.0 | 0.001541 |
| buintsC8 | 0.001465 |
| geatmaF1aC8_gma1a80.0 | 0.001452 |
| bumobsC8 | 0.001358 |
| geatmaF1aC8_gma1a81.0 | 0.001288 |
| acctypeC8_acc85.0 | 0.001188 |
| famstruct_fs5.0 | 0.000583 |
| acctypeC8_acc83.0 | 0.000391 |
| acctypeC8_acc86.0 | 0.000389 |
| famstruct_fs6.0 | 0.000309 |
| ses_ses7.0 | 0.000252 |
| acctypeC8_acc87.0 | 0.000164 |
| ses_ses8.0 | 8.474743568990274e-05 |
| acctypeC8_acc88.0 | 6.41697259511448e-05 |
| ses_ses6.0 | 3.931931039772735e-05 |

^a^ For detailed explanations of each feature code, refer to Table S3. Codes for features can be found in the 'Modalities' and 'Assessment Grade' columns.

**Table S3**

Complete list of training features for all models.

| **Features Type** | **Modalities (Feature code)^a^** | **Assessment Grade (Feature code)** | **Assessment type** |
| --- | --- | --- | --- |
| ***Family background*** |  |  |  |
| Family structure | Married spouses and their biological children (famstruct_fs1.0)  Unmarried spouses and their biological children (famstruct_fs2.0)  Spouses with children from previous relationships (famstruct_fs3.0)  Single parent with children (famstruct_fs4.0)  Other, shared parenthood after divorce/separation (famstruct_fs5.0)  Foster parent/foster home (famstruct_fs6.0) | Kindergarten, spring | Parental report |
| Type of accommodation | With mother and father (acctypeC8_acc81.0 / acctypeC9_acc91.0 / acctypeC11_acc111.0 in Grades 6, 7, and 9, respectively)  With mother (acctypeC8_acc82.0 / acctypeC9_acc92.0 / acctypeC11_acc112.0)  With father  (acctypeC8_acc83.0 / acctypeC9_acc93.0 / acctypeC11_acc113.0)  With mother and father by turns  (acctypeC8_acc84.0 / acctypeC9_acc94.0 / acctypeC11_acc114.0)  With mother and stepfather  (acctypeC8_acc85.0 / acctypeC9_acc95.0 / acctypeC11_acc115.0)  With father and stepmother  (acctypeC8_acc86.0 / acctypeC9_acc96.0 / acctypeC11_acc116.0)  With foster family or reform school  (acctypeC8_acc87.0 / acctypeC9_acc97.0 / acctypeC11_acc117.0)  With someone else  (acctypeC8_acc88.0 / acctypeC9_acc98.0 / acctypeC11_acc118.0) | Grade 6  Grade 7  Grade 9 | Parental report |
| Socio-economic status | Entrepreneurs (ses_ses1.0)  Higher white collar (ses_ses3.0)  Lower white collar  (ses_ses4.0)  Workers  (ses_ses5.0)  Students  (ses_ses6.0)  Pensioners  (ses_ses7.0)  Others  **(**ses_ses8.0**)** | Kindergarten, spring | Parental report |
| Parental education | No vocational education,  Vocational courses (min.4 months), Vocational school degree,  Vocational college degree,  Polytechnic degree or Bachelor's degree,  Master's degree,  Licentiate or Doctoral degree | Kindergarten, spring (voedo) | Parental report |
| ***Individual factors*** |  |  |  |
| Difficulty of math tasks | Easy  Relatively easy  Neutral  Relatively difficult  Difficult | Grade 6 (difmataC8)  Grade 7 (difmataC9)  Grade 9 (difmataC11) | Self-report |
| Difficulty of literacy tasks | Easy  Relatively easy  Neutral  Relatively difficult  Difficult | Grade 6 (diflitaC8)  Grade 7 (diflitaC9)  Grade 9 (diflitaC11) | Self-report |
| Difficulties in reading | No difficulties  Small difficulties  Moderate difficulties  Large difficulties | Grade 7 (diflitC9)  Grade 9 (difreaC11) | Self-report |
| Difficulties in mathematics | No difficulties  Small difficulties  Moderate difficulties  Large difficulties | Grade 7 (difmatC9)  Grade 9 (difmatC11) | Self-report |
| Absence from school, how many days after Christmas, truancy | Not at all  1-2 days  3-5 days  Over 5 days | Grade 6 (abs2C8)  Grade 7 (abs2C9)  Grade 9 (abs2C11) | Self-report |
| Self-esteem (Rosenberg's Self-Esteem Scale, shortened version) | Score | Grade 6 (rses_G6)  Grade 7 (rses_G7)  Grade 9 (rses_G9) | Self-report |
| Burn-out, exhaustion (School burnout inventory) | Score | Grade 6 (buexh_G6)  Grade 7 (buexh_G7)  Grade 9 (buexh_G11) | Self-report |
| Burn-out, cynicism (School burnout inventory) | Score | Grade 6 (bucyn_G6)  Grade 7 (bucyn_G7)  Grade 9 (bucyn_G9) | Self-report |
| Puberty onset |  | Information from Grade 6, 7, 9 (pubonset) | Self-report |
| Gender |  | Information from Kindergarten through Grade 9 (chgenderC1C13) | Self-report |
| ***Individual's behavior*** |  |  |  |
| Prosocial scale (SDQ scale) | Score | Grade 6 (prosoc_G6)  Grade 7 (prosoc_G7)  Grade 9 (prosoc_G9) | Self-report |
| Hyperactivity scale (SDQ scale) | Score | Grade 6 (hyper_G6)  Grade 7 (hyper_G7)  Grade 9 (hyper_G9) | Self-report |
| Emotional symptoms scale (SDQ scale) | Score | Grade 6 (emot_G6)  Grade 7 (emot_G7)  Grade 9 (emot_G9) | Self-report |
| Conduct problems scale (SDQ scale) | Score | Grade 6 (cprob_G6)  Grade 7 (cprob_G7)  Grade 9 (cprob_G9) | Self-report |
| Peer problems scale (SDQ scale) | Score | Grade 6 (peerp_G6)  Grade 7 (peerp_G7)  Grade 9 (peerp_G9) | Self-report |
| ***Motivation*** |  |  |  |
| Failure attributions, succeed poorly in literacy, inadequate instruction | No  Yes | Grade 6 (geatliF1aC8)  Grade 7 (geatliF1aC9)  Grade 9 (geatliF1aC11) | Self-report |
| Failure attributions, succeed poorly in literacy, don't try enough | No  Yes | Grade 6 (geatliF1bC8)  Grade 7 (geatliF1bC9)  Grade 9 (geatliF1bC11) | Self-report |
| Failure attributions, succeed poorly in literacy, not skillful enough | No  Yes | Grade 6 (geatliF1cC8)  Grade 7 (geatliF1cC9)  Grade 9 (geatliF1cC11) | Self-report |
| Failure attributions, succeed poorly in literacy, difficult tasks | No  Yes | Grade 6 (geatliF1dC8)  Grade 7 (geatliF1dC9)  Grade 9 (geatliF1dC11) | Self-report |
| Failure attributions, succeed poorly in mathematics, inadequate instruction | No  Yes | Grade 6 (geatmaF1aC8)  Grade 7 (geatmaF1aC9)  Grade 9 (geatmaF1aC11) | Self-report |
| Failure attributions, succeed poorly in mathematics, don't try enough | No  Yes | Grade 6 (geatmaF1bC8)  Grade 7 (geatmaF1bC9)  Grade 9 (geatmaF1bC11) | Self-report |
| Failure attributions, succeed poorly in mathematics, not skillful enough | No  Yes | Grade 6 (geatmaF1cC8)  Grade 7 (geatmaF1cC9)  Grade 9 (geatmaF1cC11) | Self-report |
| Failure attributions, succeed poorly in mathematics, difficult tasks | No  Yes | Grade 6 (geatmaF1dC8)  Grade 7 (geatmaF1dC9)  Grade 9 (geatmaF1dC11) | Self-report |
| Failure attributions, don't know how to do a literacy task, inadequate instruction | No  Yes | Grade 6 (geatliF2aC8)  Grade 7 (geatliF2aC9)  Grade 9 (geatliF2aC11) | Self-report |
| Failure attributions, don't know how to do a literacy task, doesn't try enough | No  Yes | Grade 6 (geatliF2bC8)  Grade 7 (geatliF2bC9)  Grade 9 (geatliF2bC11) | Self-report |
| Failure attributions, don't know how to do a literacy task, not skillful enough | No  Yes | Grade 6 (geatliF2cC8)  Grade 7 (geatliF2cC9)  Grade 9 (geatliF2cC11) | Self-report |
| Failure attributions, don't know how to do a literacy task, difficult tasks | No  Yes | Grade 6 (geatliF2dC8)  Grade 7 (geatliF2dC9)  Grade 9 (geatliF2dC11) | Self-report |
| Failure attributions, don't know how to do a math task, inadequate instruction | No  Yes | Grade 6 (geatmaF2aC8)  Grade 7 (geatmaF2aC9)  Grade 9 (geatmaF2aC11) | Self-report |
| Failure attributions, don't know how to do a math task, doesn't try enough | No  Yes | Grade 6 (geatmaF2bC8)  Grade 7 (geatmaF2bC9)  Grade 9 (geatmaF2bC11) | Self-report |
| Failure attributions, don't know how to do a math task, not skillful enough | No  Yes | Grade 6 (geatmaF2cC8)  Grade 7 (geatmaF2cC9)  Grade 9 (geatmaF2cC11) | Self-report |
| Failure attributions, don't know how to do a math task, difficult tasks | No  Yes | Grade 6 (geatmaF2dC8)  Grade 7 (geatmaF2dC9)  Grade 9 (geatmaF2dC11) | Self-report |
| Self-concept literacy (mean general & compared to others) | Score | Grade 6 (selfli_G6)  Grade 7 (selfli_G7)  Grade 9 (selfli_G9) | Self-report |
| Self-concept math (mean general & compared to others) | Score | Grade 6 (selfma_G6)  Grade 7 (selfma_G7)  Grade 9 (selfma_G9) | Self-report |
| Task value, literacy (mean importance, interest, utility) | Score | Grade 6 (tavli_G6)  Grade 7 (tavli_G7)  Grade 9 (tavli_G9) | Self-report |
| Task value, math (mean importance, interest, utility) | Score | Grade 6 (tavma_G6)  Grade 7 (tavma_G7)  Grade 9 (tavma_G9) | Self-report |
| To what extent do you know your plans after comprehensive education | My plans after comprehensive school are still completely unclear.  I am still uncertain of my further plans after comprehensive school.  I know exactly what I will do after comprehensive school. | Grade 7 (plan_aC9rec)  Grade 9 (plan_aC11rec) | Self-report |
| ***Engagement*** |  |  |  |
| School belonging | Score | Grade 7 (schbe_G7)  Grade 9 (schbe_G9) | Self-report |
| Class engagement | Score | Grade 7 (claeng_G7)  Grade 9 (claeng_G9) | Self-report |
| Behavioral engagement (Research Assessment Package for Schools) | Score | Grade 6 (behen_G6)  Grade 7 (behen_G7)  Grade 9 (behen_G9) | Self-report |
| Teacher–student relationships (Student Engagement Instrument) | Score | Grade 7 (testre_G7)  Grade 9 (testre_G9) | Self-report |
| Control and relevance of school work (Student Engagement Instrument) | Score | Grade 7 (schwo_G7)  Grade 9 (schwo_G9) | Self-report |
| Peer support for learning (Student Engagement Instrument) | Score | Grade 7 (peersup_G7)  Grade 9 (peersup_G9) | Self-report |
| Future aspirations and goals (Student Engagement Instrument) | Score | Grade 7 (future_G7)  Grade 9 (future_G9) | Self-report |
| Family support for learning (Student Engagement Instrument) | Score | Grade 7 (familysup_G7)  Grade 9 (familysup_G9) | Self-report |
| ***Bullying*** |  |  |  |
| "How often have you been bullied at school after Christmas? frequency | I haven’t been bullied  Once or twice  2 or 3 times in month  Once a week  Several times a week  Can’t say | Grade 6 (bullfrC8)  Grade 7 (bullfrC9)  Grade 9 (bullfrC11) | Self-report |
| "How many times have you bullied another student at school after Christmas?", bullies him/herself | I haven’t bullied anyone  Once or twice  2 or 3 times in month  Once a week  Several times a week | Grade 6 (bullseC8)  Grade 7 (bullseC9)  Grade 9 (bullseC11) | Self-report |
| Bullying by mobile phone, "have you been bullied..." | I haven’t been bullied  Once or twice  2 or 3 times per month  Approximately once a week  Several times a week | Grade 6 (bumobC8)  Grade 7 (bumobC9)  Grade 9 (bumobC11) | Self-report |
| Bullying on internet, "have you been bullied..." | I haven’t been bullied Once or twice  2 or 3 times per month  Approximately once a week  Several times a week | Grade 6 (buintC8)  Grade 7 (buintC9)  Grade 9 (buintC11) | Self-report |
| Bullying by mobile phone "have you bullied...self" | I haven’t bullied  Once or twice  2 or 3 times per month  Approximately once a week  Several times a week | Grade 6 (bumobsC8)  Grade 7 (bumobsC9)  Grade 9 (bumobsC11) | Self-report |
| Bullying on internet, "have you bullied...self" | I haven’t bullied  Once or twice  2 or 3 times per month  Approximately once a week  Several times a week | Grade 6 (buintsC8)  Grade 7 (buintsC9)  Grade 9 (buintsC11) | Self-report |
| ***Health behavior*** |  |  |  |
| Smoking | Never  Tried  Sometimes (less than once a week)  Once or more per week – not daily  Daily | Grade 6 (hesmoC8)  Grade 7 (hesmoC9)  Grade 9 (hesmoC11) | Self-report |
| Alcohol use | Never  Tried  Sometimes (less than once per month)  Twice a month  Once a week or more | Grade 6 (healcC8)  Grade 7 (healcC9)  Grade 9 (healcC11) | Self-report |
| ***Media usage*** |  |  |  |
| Use of media, listening to music | Not at all  Less than an hour  1-2 hours  3-4 hours  More than 4 hours | Grade 6 (med4C8) | Self-report |
| Use of media, listening radio | Not at all  Less than an hour  1-2 hours  3-4 hours  More than 4 hours | Grade 6 (med5C8) | Self-report |
| Use of media, television | Not at all  Less than an hour  1-2 hours  3-4 hours  More than 4 hours | Grade 6 (med1C8)  Grade 7 (med1C9)  Grade 9 (med1C11) | Self-report |
| Use of media, on computer or internet on mobile phone | Not at all  Less than an hour  1-2 hours  3-4 hours  More than 4 hours | Grade 6 (med2C8)  Grade 7 (med2C9)  Grade 9 (med2C11) | Self-report |
| Use of media, mobile phone (calling, texting) | Not at all  Less than an hour  1-2 hours  3-4 hours  More than 4 hours | Grade 6 (med3C8)  Grade 7 (med3C9)  Grade 9 (med3C11) | Self-report |
| Use of media, game console | Not at all  Less than an hour  1-2 hours  3-4 hours  More than 4 hours | Grade 7 (med4C9)  Grade 9 (med4C11) | Self-report |
| Use of mobile phone, sending SMS, WhatsApp or KIK messages (private) | Not at all  Some uses per week  1-10 uses per day  11-20 uses per day  20-50 uses per day  Over 50 uses per day | Grade 6 (mob1C8)  Grade 7 (mob1C9)  Grade 9 (mob1C11) | Self-report |
| Use of mobile phone, sending attachments | Not at all  Some uses per week  1-10 uses per day  11-20 uses per day  20-50 uses per day  Over 50 uses per day | Grade 6 (mob2C8) | Self-report |
| Use of mobile phone, sending public updates using e.g., Instagram, Facebook, Twitter | Not at all  Some uses per week  1-10 uses per day  11-20 uses per day  20-50 uses per day  Over 50 uses per day | Grade 9 (mob1pubC11) | Self-report |
| Use of Internet, using e-mail | Never  Once or twice per month  Once a week  Few times a week daily | Grade 6 (inter1C8)  Grade 7 (inter1C9)  Grade 9 (inter1C11) | Self-report |
| Use of Internet, surfing or browsing websites | Never  Once or twice per month  Once a week  Few times a week daily | Grade 6 (inter2C8)  Grade 7 (inter2C9)  Grade 9 (inter2C11) | Self-report |
| Use of Internet, seeking information | Never  Once or twice per month  Once a week  Few times a week daily | Grade 6 (inter3C8)  Grade 7 (inter3C9)  Grade 9 (inter3C11) | Self-report |
| Use of Internet, watching videos (e.g., on YouTube) | Never  Once or twice per month  Once a week  Few times a week daily | Grade 6 (inter4C8)  Grade 7 (inter4C9)  Grade 9 (inter4C11) | Self-report |
| Use of Internet, listening to music | Never  Once or twice per month  Once a week  Few times a week daily | Grade 6 (inter5C8)  Grade 7 (inter5C9)  Grade 9 (inter5C11) | Self-report |
| Use of Internet, blogging | Never  Once or twice per month  Once a week  Few times a week daily | Grade 6 (inter6C9)  Grade 7 (inter6C9)  Grade 9 (inter6C11) | Self-report |
| Use of Internet, upholding home pages | Never  Once or twice per month  Once a week  Few times a week daily | Grade 6 (inter7C8)  Grade 7 (inter7C9)  Grade 9 (inter7C11) | Self-report |
| Use of Internet, downloading files | Never  Once or twice per month  Once a week  Few times a week daily | Grade 6 (inter8C8)  Grade 7 (inter8C9)  Grade 9 (inter8C11) | Self-report |
| Use of Internet, playing computer or console games | Never  Once or twice per month  Once a week  Few times a week daily | Grade 6 (inter9C8)  Grade 7 (inter9C9)  Grade 9 (inter9C11) | Self-report |
| Use of Internet, playing or creating characters with others | Never  Once or twice per month  Once a week  Few times a week daily | Grade 6 (inter10C8)  Grade 7 (inter10C9)  Grade 9 (inter10C11) | Self-report |
| Use of Internet, playing site games | Never  Once or twice per month  Once a week  Few times a week daily | Grade 6 (inter11C8)  Grade 7 (inter11C9)  Grade 9 (inter11C11) | Self-report |
| Use of Internet, playing on mobile device (e.g., Angry Birds, Temple Run, Subway Surfers) | Never  Once or twice per month  Once a week  Few times a week daily | Grade 6 (inter12C8)  Grade 7 (inter12C9)  Grade 9 (inter12C11) | Self-report |
| Use of Internet, skyping | Never  Once or twice per month  Once a week  Few times a week daily | Grade 6 (inter13C8)  Grade 7 (inter13C9)  Grade 9 (inter13C11) | Self-report |
| Use of Internet, using Instagram | Never  Once or twice per month  Once a week  Few times a week daily | Grade 6 (inter14C8)  Grade 7 (inter14C9)  Grade 9 (inter14C11) | Self-report |
| Use of Internet, Periscope | Never  Once or twice per month  Once a week  Few times a week daily | Grade 9 (inter15C11) | Self-report |
| Use of Internet, Twitter | Never  Once or twice per month  Once a week  Few times a week daily | Grade 6 (inter14C8)  Grade 7 (inter14C9)  Grade 9 (inter16C11) | Self-report |
| Use of Internet, Facebook | Never  Once or twice per month  Once a week  Few times a week daily | Grade 6 (inter15C8)  Grade 7 (inter15C9)  Grade 9 (inter17C11) | Self-report |
| Use of Internet, taking part in Habbo or other online communities | Never  Once or twice per month  Once a week  Few times a week daily | Grade 6 (inter16C8)  Grade 7 (inter16C9) | Self-report |
| Use of Internet, using Instant message program | Never  Once or twice per month  Once a week  Few times a week daily | Grade 6 (inter17C8)  Grade 7 (inter17C9) | Self-report |
| Use of Internet, Pinterest | Never  Once or twice per month  Once a week  Few times a week daily | Grade 9 (inter18C11) | Self-report |
| Use of Internet, Ask.fm | Never  Once or twice per month  Once a week  Few times a week daily | Grade 9 (inter19C11) | Self-report |
| Use of Internet, reading blogs | Never  Once or twice per month  Once a week  Few times a week daily | Grade 6 (inter18C8)  Grade 7 (inter18C9)  Grade 9 (inter20C11) | Self-report |
| Use of Internet, visit forums | Never  Once or twice per month  Once a week  Few times a week daily | Grade 9 (inter21C11) | Self-report |
| Use of Internet, playing learning games | Never  Once or twice per month  Once a week  Few times a week daily | Grade 6 (inter19C8)  Grade 7 (inter19C9)  Grade 9 (inter22C11) | Self-report |
| Use of Internet, reading online magazines | Never  Once or twice per month  Once a week  Few times a week daily | Grade 6 (inter20C8)  Grade 7 (inter20C9)  Grade 9 (inter23C11) | Self-report |
| Use of Internet, online shopping | Never  Once or twice per month  Once a week  Few times a week daily | Grade 6 (inter21C8)  Grade 7 (inter21C9)  Grade 9 (inter24C11) | Self-report |
| Use of Internet, using imaging software | Never  Once or twice per month  Once a week  Few times a week daily | Grade 6 (inter22C8)  Grade 7 (inter22C9)  Grade 9 (inter28C11) | Self-report |
| Use of Internet, editing videos | Never  Once or twice per month  Once a week  Few times a week daily | Grade 6 (inter23C8)  Grade 7 (inter23C9)  Grade 9 (inter26C11) | Self-report |
| Use of Internet, making animations or 3D modeling | Never  Once or twice per month  Once a week  Few times a week daily | Grade 6 (inter24C8)  Grade 7 (inter24C9)  Grade 9 (inter27C11) | Self-report |
| Use of Internet, coding (e.g. code.org, scratch) | Never  Once or twice per month  Once a week  Few times a week daily | Grade 9 (inter28C11) | Self-report |
| Use of Internet, making music | Never  Once or twice per month  Once a week  Few times a week daily | Grade 6 (inter25C8)  Grade 7 (inter25C9)  Grade 9 (inter29C11) | Self-report |
| Use of Internet, filling in inquiries or participating in competitions | Never  Once or twice per month  Once a week  Few times a week daily | Grade 6 (inter26C8)  Grade 7 (inter26C9)  Grade 9 (inter30C11) | Self-report |
| ***Cognitive skills*** |  |  |  |
| Rapid serial naming of objects | Score | Kindergarten, spring (RAN) | Assessment |
| Counting | Score | Kindergarten, spring (msqSC2) | Assessment |
| Vocabulary (Peabody Picture Vocabulary Test - Revised) | Score | Kindergarten, spring (ppvSC2) | Assessment |
| Arithmetic fluency | Score | kindergarten, spring (ariSC2) | Assessment |
| Raven | Score | Grade 3 (ravSC6) | Assessment |
| ***Academic outcomes*** |  |  |  |
| Reading fluency | Score | Grade 1 (READ1)  Grade 2 (READ2) Grade 3 (READ3)  Grade 4 (READ4)  Grade 6 (READ6)  Grade 7 (READ7)  Grade 9 (READ9) | Assessment |
| Reading comprehension | Score | Grade 1 (ly1C4c)  Grade 2 (ly1C5c)  Grade 3 (ly1C6c)  Grade 4 (ly4C7c)  Grade 6 (ly6C8c)  Grade 7 (ly7C9c)  Grade 9 (ly7C11c) | Assessment |
| PISA score | Score | Grade 9 (pisaC10total_sum) | Assessment |
| Arithmetic fluency | Score | Grade 1, fall (ariSC3)  Grade 1, spring (ariSC4)  Grade 2 (ariSC5)  Grade 3 (ariSC6)  Grade 4 (ariSC7)  Grade 6 (ariSC8)  Grade 7 (ariSC9)  Grade 9 (ariSC11) | Assessment |
| Multiplication | Score | Grade 4 (multSC7)  Grade 6 (multSC8)  Grade 7 (multSC9)  Grade 9 (multSC11) | Assessment |

^a^ For the features family structure, accommodation type, and socio-economic status, feature codes are listed in the 'Modalities' column. For the rest, codes are provided in the 'Assessment Grade' column.

**Supplementary Note**

**Educational system in Finland**

In Finland, children commence pre-primary education in August when children turn six. Subsequently, at the age of seven, they enter the 9-year comprehensive school, which encompasses primary school (Grades 1–6) and lower secondary school (Grades 7–9). Following this phase, students apply in a joint application process to enroll in upper secondary education, typically opting for either a general upper secondary school (academic track) or a vocational school (vocational track). During the data collection period, 94% of grade-9 students applied for upper secondary education (Statistics Finland, 2022). Since 2021, upper secondary education has become mandatory for all individuals under the age of 18. However, students retain the flexibility to switch between tracks and alter their chosen subjects within these tracks after the commencement of secondary education.
